# A Novel Zinc(II) Complex for Sonodynamic Therapy Induces Pyroptosis of Breast Cancer Cells and Enhances Anti‐Tumor Immune Response

**DOI:** 10.1002/advs.202508155

**Published:** 2025-10-21

**Authors:** Sijie Wen, Dongsheng Tang, Lingpu Zhang, Hanchen Zhang, Zheng Cao, Kong Wai Tan, Xiaojun Wang, Haihua Xiao, Kun Shang

**Affiliations:** ^1^ Beijing National Laboratory for Molecular Sciences Laboratory of Polymer Physics and Chemistry Institute of Chemistry Chinese Academy of Sciences Beijing 100190 P. R. China; ^2^ University of Chinese Academy of Sciences Beijing 100049 P. R. China; ^3^ Department of Chemical and Biomolecular Engineering University of California Los Angeles CA 90095 USA; ^4^ Department of Chemistry Faculty of Science Universiti Malaya Kuala Lumpur 50603 Malaysia; ^5^ Department of Ophthalmology Beijing Chaoyang Hospital Capital Medical University Beijing 100020 P. R. China; ^6^ Department of Nuclear Medicine Peking University People's Hospital Beijing 100044 P. R. China

**Keywords:** breast cancer, pyroptosis, sonodynamic therapy, zinc(II) complex

## Abstract

Zinc(II) complexes have emerged as promising antitumor agents due to their favorable biosafety and biocompatibility, and demonstrated effectiveness in photodynamic therapy (PDT). However, their therapeutic potential is limited by the poor tissue penetration of PDT agents. Herein, a novel zinc(II) complex designed for sonodynamic therapy (SDT), capable of producing substantial amounts of singlet oxygen upon ultrasonic activation, is introduced. This sonosensitizer effectively induces pyroptosis in breast cancer cells, significantly promoting the antitumor immune response. This work represents the first exploration of zinc(II) complexes in SDT, establishing a new avenue for enhanced therapeutic outcomes against breast cancer.

## Graphical abstract

Although Zinc(II) complexes are effective in photodynamic therapy, their efficacy is limited by the poor tissue penetration of light. This work introduces a novel zinc(II) complex for sonodynamic therapy. Under ultrasound activation, it generates substantial singlet oxygen, induces pyroptosis in breast cancer cells, and promotes anti‐tumor immune response, establishing a new avenue for enhanced therapeutic outcomes against breast cancer.

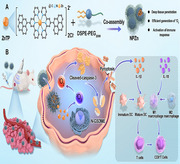

## 1 Introduction

In recent years, significant advancements have been achieved in the development of transition metal complexes as anticancer pharmaceuticals, leading to various novel transition metal complex drugs that exhibit low toxicity and high anticancer efficacy. Notably, zinc(II) complexes have gained considerable attention owing to their exceptional biosafety and biocompatibility profiles.^[^ ^1^ ^]^ As the second most abundant trace metal element in the human body, zinc rarely exists in free ionic form. Instead, it predominantly occurs as zinc(II) complexes, which play pivotal roles in critical biological processes, including cellular constitution, signal transduction, and enzymatic catalysis. Furthermore, they have exhibited minimal adverse effects as anticancer therapeutics.^[^ ^2^, ^3^, ^4^ ^]^ Currently, various zinc(II) complexes have been synthesized. Among them, phthalocyanine‐zinc(II) complexes, serving as photosensitizers, have demonstrated remarkable efficacy in photodynamic therapy (PDT).^[^ ^5^, ^6^, ^7^, ^8^, ^9^ ^]^ However, the limited tissue penetration depth of PDT restricts the therapeutic efficacy of zinc(II) complexes in the treatment of deep‐seated tumors. With the emergence of sonodynamic therapy (SDT), the issue of poor tissue penetration in PDT has been effectively addressed.^[^ ^10^, ^11^, ^12^ ^]^ SDT employs low‐intensity therapeutic ultrasound (1.0 MHz, 0.5–2.5 W cm^−2^) to activate sonosensitizers, generating reactive oxygen species (ROS).^[^ ^13^, ^14^ ^]^ The antitumor effect of SDT hinges on the efficiency of ROS production by the sonosensitizers. Although common sonosensitizers (such as Ce6 and ICG) have achieved certain successes, their efficiency in generating ROS under ultrasound remains to be improved.^[^ ^15^ ^,^ ^16^ ^]^

**Scheme 1:**
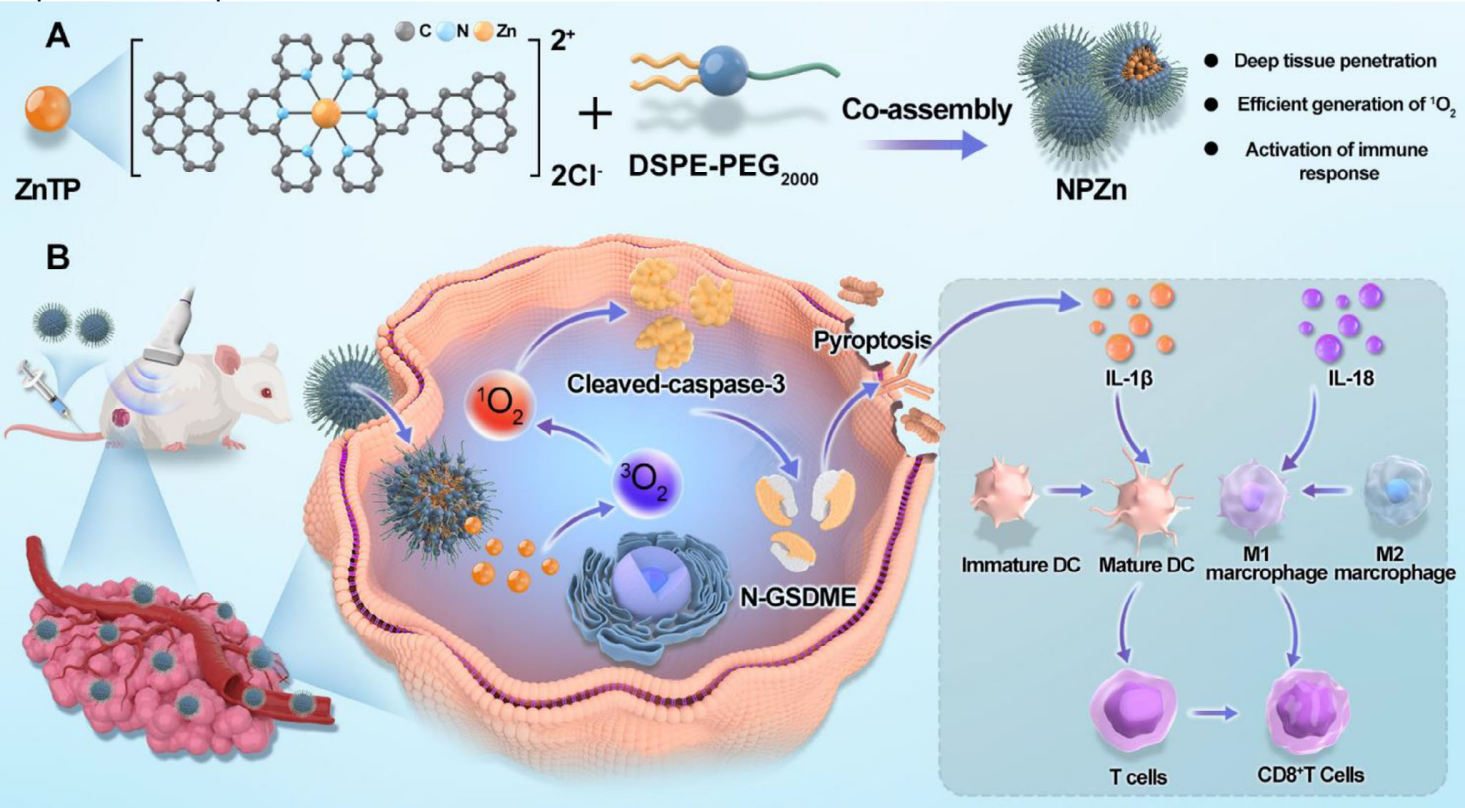
Schematic diagram of a new zinc (II) sonosensitizer for the treatment of breast cancer. A) Schematic diagram of the zinc(II) complex designed in this work. ZnTP is further co‐assembled with DSPE‐PEG_2000_ to form NPZn. B) The antitumor mechanism of NPZn. After intravenous (*i.v*.) injection, NPZn accumulates at the tumor site. Upon exposure to ultrasound, NPZn generates a significant amount of singlet oxygen (^1^O_2_), which subsequently triggers pyroptosis in cancer cells. Additionally, the process leads to the production of inflammatory factors, activating the immune response and ultimately inhibiting the progression of breast cancer.

Due to the robust ROS‐generating capability of zinc(II) complexes as photosensitizers and the limited availability of reports on zinc(II) complexes functioning as sonosensitizers, our efforts have been directed toward developing a novel zinc(II) complex for SDT. Herein, a zinc(II) complex, ZnTP, featuring a zinc atom center and two ligands consisting of terpyridine and pyrene (TP), is first reported (**Scheme** **1**A). Under ultrasound irradiation, ZnTP generates a substantial amount of singlet oxygen (^1^O_2_), subsequently inducing pyroptosis in breast cancer cells. Furthermore, ZnTP was co‐assembled with the commercial amphiphilic polymer DSPE‐PEG_2000_ to form nanoparticles (NPZn), which effectively accumulate at tumor sites in mice after intravenous (*i.v*.) administration. Under ultrasound irradiation, NPZn not only induces pyroptosis but also activates and enhances antitumor immune response (Scheme 1B).

## 2 Results and Discussion

**Figure 1:**
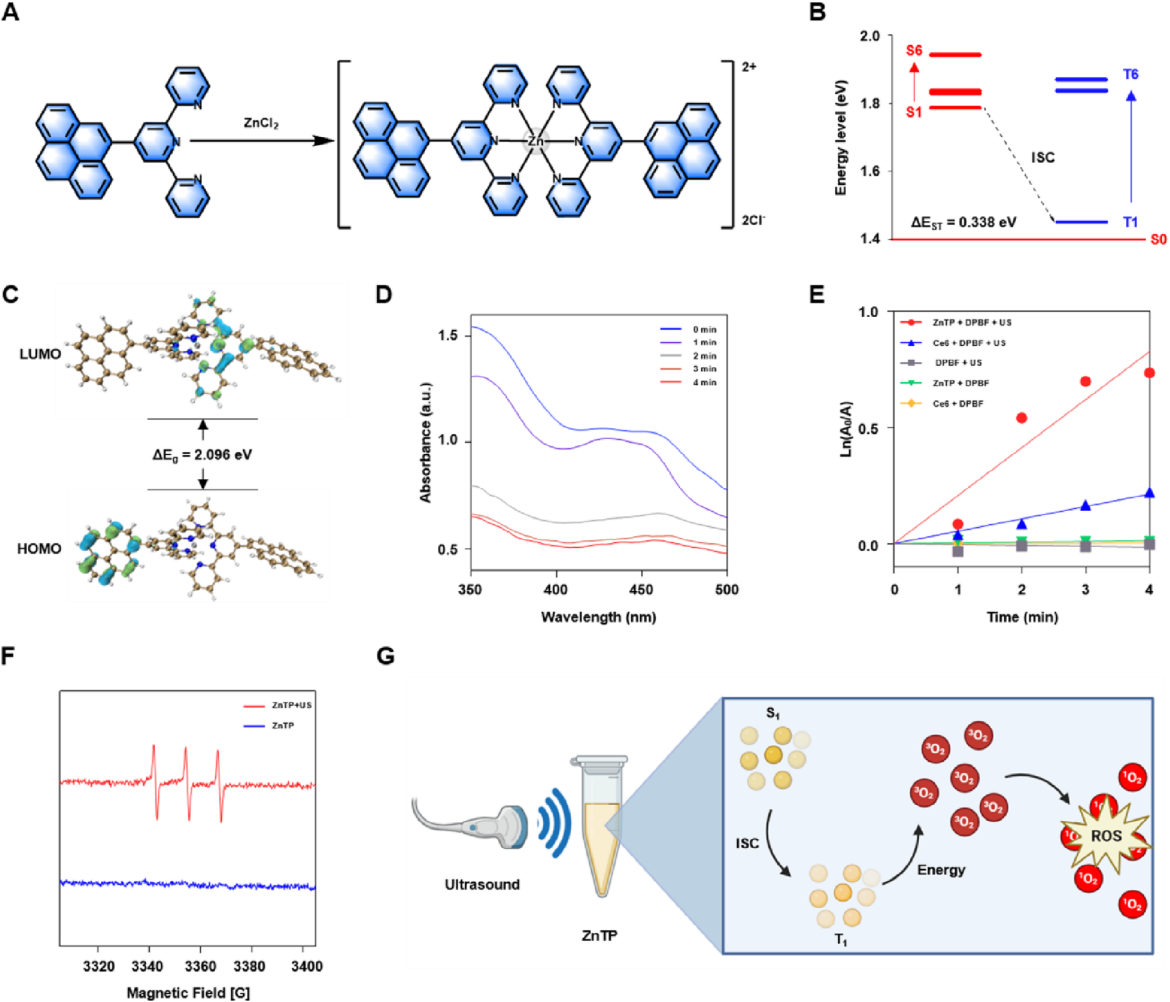
Structure, preparation, and sonodynamic performance of ZnTP. A) Synthetic route of ZnTP. B) After structural optimization, the S_1_‐S_6_ and T_1_‐T_6_ energy levels of ZnTP and the energy gap between S_1_ and T_1_ (ΔE_ST_) were calculated through vertical excitation. C) The distribution of HOMO‐LUMO and the energy gap (ΔE_g_) of ZnTP. D) Absorption spectra of 1,3‐diphenylisobenzofuran (DPBF) with ZnTP over time under ultrasound irradiation. E) The plot of ln(A_0_/A) upon exposure to ultrasound irradiation. F) Electron spin resonance spectrum of ^1^O_2_ generated by ZnTP under ultrasound irradiation. G) Mechanism of reactive oxygen species (ROS) production by ZnTP under ultrasound irradiation. Ultrasonic conditions in (D–F): 1.0 MHz, 1.0 W cm^−2^, 50% duty cycle, 2 min.

The ligand TP was synthesized through a Suzuki coupling reaction between 4′‐bromo‐2,2′:6′,2′'‐terpyridine and 4,4,5,5‐tetramethyl‐2‐(pyren‐1‐yl)‐1,3,2‐dioxaborolane, followed by purification via column chromatography. TP was further coordinated with zinc chloride (ZnCl_2_) to obtain ZnTP (**Figure** **1**A; Figure S1, Supporting Information). The final product was characterized by ^1^H‐nuclear magnetic resonance (^1^H‐NMR) and electrospray ionization Fourier transform ion cyclotron resonance mass spectrometry (ESI‐FTICR‐MS) (Figures S2–S5, Supporting Information). The photophysical properties of ZnTP were characterized by UV–vis spectroscopy (Figure S6, Supporting Information), revealing a strong absorption peak at 349 nm. Due to the crucial role of ROS in SDT, the ability of ZnTP to generate ROS was first predicted using density functional theory (DFT) calculations. As shown in Figure 1B, ZnTP exhibits an energy gap (ΔE_ST_) between the lowest singlet (S_1_) and triplet (T_1_) states of 0.338 eV. A smaller ΔE_ST_ can enhance the rate of intersystem crossing, thereby improving ZnTP's ability to generate ROS.^[^ ^17^ ^]^ In addition, ZnTP has an energy gap (ΔE_g_) of 2.096 eV between the highest occupied molecular orbital (HOMO) and lowest unoccupied molecular orbital (LUMO) (Figure 1C). This moderate ΔE_g_ ensures effective separation of electrons and holes while making the molecule relatively easy to excite, facilitating ROS production. The DFT results indicate that ZnTP possesses excellent ROS‐generating ability due to its small ΔE_ST_ and moderate ΔE_g_.^[^ ^18^, ^19^ ^]^ Therefore, ZnTP has potential for application in SDT.

To investigate the ROS generation capability of ZnTP as a sonosensitizer, 1,3‐diphenylisobenzofuran (DPBF) was employed as a probe. Under ultrasound irradiation (1.0 MHz, 1.0 W cm^−2^, 50% duty cycle), a significant decrease in the absorbance of DPBF at 410 nm was observed. This result demonstrates that ZnTP can generate ROS under ultrasound irradiation, leading to the decomposition of DPBF (Figure 1D). Subsequently, the ROS generation efficiency of ZnTP was evaluated and compared with the standard sonosensitizer Ce6. As shown in Figure 1E, ZnTP could produce more ROS compared to Ce6, further demonstrating its potential as an effective sonosensitizer. Next, the sources of ROS generated by ZnTP as a sonosensitizer were analyzed. Generally, the sources of ROS involve two pathways: Type I from electron transfer, and Type II from energy transfer. The Type I process produces superoxide anions, which can further be converted into hydroxyl radicals. In comparison, the Type II process directly generates ^1^O_2_.^[^ ^20^, ^21^ ^]^

To investigate whether ZnTP generates ROS through the Type I process, methylene blue (MB) was employed as a hydroxyl radical scavenger, and its absorbance at a wavelength of 660 nm was monitored under ultrasound irradiation (1.0 MHz, 1.0 W cm^−2^, 50% duty cycle). Experimental results indicated that during the 4 min ultrasound treatment, no significant change in the absorbance of MB was observed, suggesting that ZnTP does not produce hydroxyl radicals through the Type I process (Figure S7, Supporting Information). To determine whether ZnTP generates ROS through a Type II process, the effects of ZnTP on ROS generation under ultrasound irradiation were investigated in the presence of either furfuryl alcohol, a singlet oxygen scavenger, or p‐benzoquinone, a superoxide anion scavenger. It was observed that ROS production efficiency was significantly reduced in the presence of furfuryl alcohol, whereas no inhibitory effect was found with p‐benzoquinone. These results demonstrate that ^1^O_2_ is specifically generated by ZnTP via a Type II mechanism (Figures S8 and S9, Supporting Information). Subsequently, electron spin resonance (ESR) spectroscopy was used to detect the production of ^1^O_2_ by ZnTP, with 2,2,6,6‐tetramethylpiperidine (TEMP) serving as the trapping agent. Compared to ZnTP without ultrasound treatment, ZnTP + US exhibited three distinct triple peak signals on the ESR spectrum. The result shows that ZnTP is capable of generating ^1^O_2_ under ultrasound irradiation (Figure 1F). The aforementioned results demonstrate that ZnTP functions as an efficient sonosensitizer, with the source of its ROS being ^1^O_2_ generated through the Type II process (Figure 1G).

**Figure 2:**
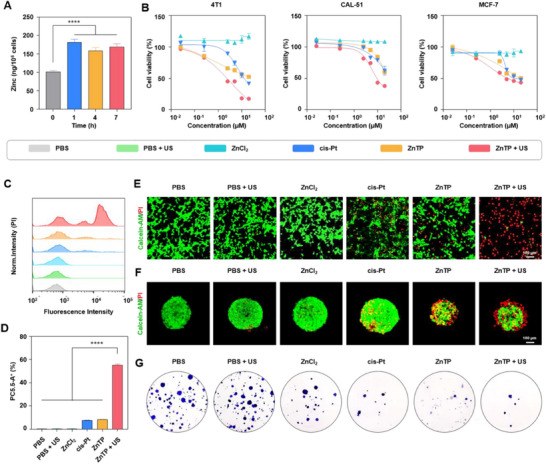
ZnTP exhibits exceptional in vitro anticancer activity under ultrasound irradiation. A) The Zn concentration in 4T1 cells after various incubation times was determined by ICP. B) Cell viability of 4T1, CAL‐51, and MCF‐7 cancer cells subjected to different treatments was assessed using the MTT assay. Ultrasonic conditions: 1.0 MHz, 1.2 W cm^−2^, 50% duty cycle, 2 min. C) Flow cytometry (FCM) was employed to analyze the uptake of propidium iodide (PI) in 4T1 cells following different treatments. Ultrasonic conditions: 1.0 MHz, 1.0 W cm^−2^, 50% duty cycle, 1 min. D) Quantitative results of PI fluorescence intensity in 4T1 cells after different treatments. E) confocal laser scanning microscopy (CLSM) images of 4T1 cells stained with Calcein‐AM (green, live cells) and PI (red, dead cells). Scale bar: 100 µm. Ultrasonic conditions: 1.0 MHz, 1.0 W cm^−2^, 50% duty cycle, 0.5 min. F) CLSM images of 4T1 tumor spheroids stained with Calcein‐AM and PI. Scale bar: 100 µm. Ultrasonic conditions: 1.0 MHz, 1.0 W cm^−2^, 50% duty cycle, 0.5 min. G) 4T1 cell colonies stained with crystal violet after different treatments. Ultrasonic conditions: 1.0 MHz, 1.0 W cm^−2^, 50% duty cycle, 1 min. The data were analyzed by one‐way ANOVA. ^****^ *p*< 0.0001. Data are presented as mean values ± SD (n = 3).

Encouraged by these results, the anticancer efficacy of ZnTP was first evaluated in vitro. Given that the prerequisite for the therapeutic agent to exert its anticancer effect is its uptake by cancer cells, the efficacy of ZnTP uptake by cancer cells was examined. First, the intracellular zinc concentration in 4T1 (mouse breast cancer) cells was quantitatively analyzed using inductively coupled plasma optical emission spectrometry (ICP‐OES). Notably, zinc is the second most common transition metal element in the human body. Therefore, an average zinc concentration of 101 ng per 10^6^ 4T1 cells was detected in the absence of ZnTP incubation.^[^ ^22^ ^]^ After 1 h of co‐incubation of ZnTP with 4T1 cells, the average zinc concentration per 10^6^ 4T1 cells markedly increased to 182 ng. This result indicates that ZnTP can be effectively taken up by cancer cells (**Figure** **2**A). Second, the cytotoxicity of ZnTP toward 4T1, CAL‐51 (human breast cancer), and MCF‐7 (human breast cancer) cells under various conditions was investigated using the 3‐(4,5‐Dimethylthiazol‐2‐yl)‐2,5‐diphenyltetrazolium bromide (MTT) assay. The results indicated that ZnTP exhibited considerable cytotoxicity against these cell lines. Under ultrasound irradiation (1.0 MHz, 1.2 W cm^−2^, 50% duty cycle, 2 min), the half‐maximal inhibitory concentration (IC_50_) of ZnTP against 4T1 cells was ≈4 µM, which was lower than the IC_50_ of cisplatin (cis‐Pt). This finding suggested that ultrasound enhanced the toxic effect of ZnTP on cancer cells. Notably, ZnCl_2_, which shares the same anion with ZnTP, exhibited negligible cytotoxicity, further validating that ZnTP possesses a different cellular action mechanism compared to zinc ions alone (Figure 2B).

To further investigate the mechanism of cell death, flow cytometry (FCM) was utilized with PI staining (Figure 2C). The results demonstrated that ZnTP exhibited a toxic effect similar to that of cis‐Pt, leading to ≈7% uptake of PI. When combined with ultrasound (1.0 MHz, 1.0 W cm^−2^, 50% duty cycle, 1 min), the proportion of ZnTP‐treated cell uptake of PI increased further to 55.2% (Figure 2D). Subsequently, the anticancer efficacy of ZnTP was investigated using confocal laser scanning microscopy (CLSM) (Figure 2E,F). Calcein‐AM (green fluorescence, representing live cells) and PI (red fluorescence, representing dead cells) staining were applied to both 2D cell cultures and 3D cell spheroids. The results revealed that the addition of ZnCl_2_ or ultrasound alone (1.0 MHz, 1.0 W cm^−2^, 50% duty cycle, 0.5 min) had minimal impact on cell viability. However, upon the addition of ZnTP and application of the same ultrasound conditions, the cells showed intense red fluorescence but weak green fluorescence, indicating more significant cell death. Finally, the anti‐proliferative ability of ZnTP was evaluated through a colony formation assay (Figure 2G). After ultrasound treatment, the number of cell colonies treated with ZnTP decreased significantly, suggesting the long‐term anticancer effects of ZnTP. Collectively, these results indicate that ZnTP exhibits excellent SDT efficacy and induces cancer cell death.

**Figure 3:**
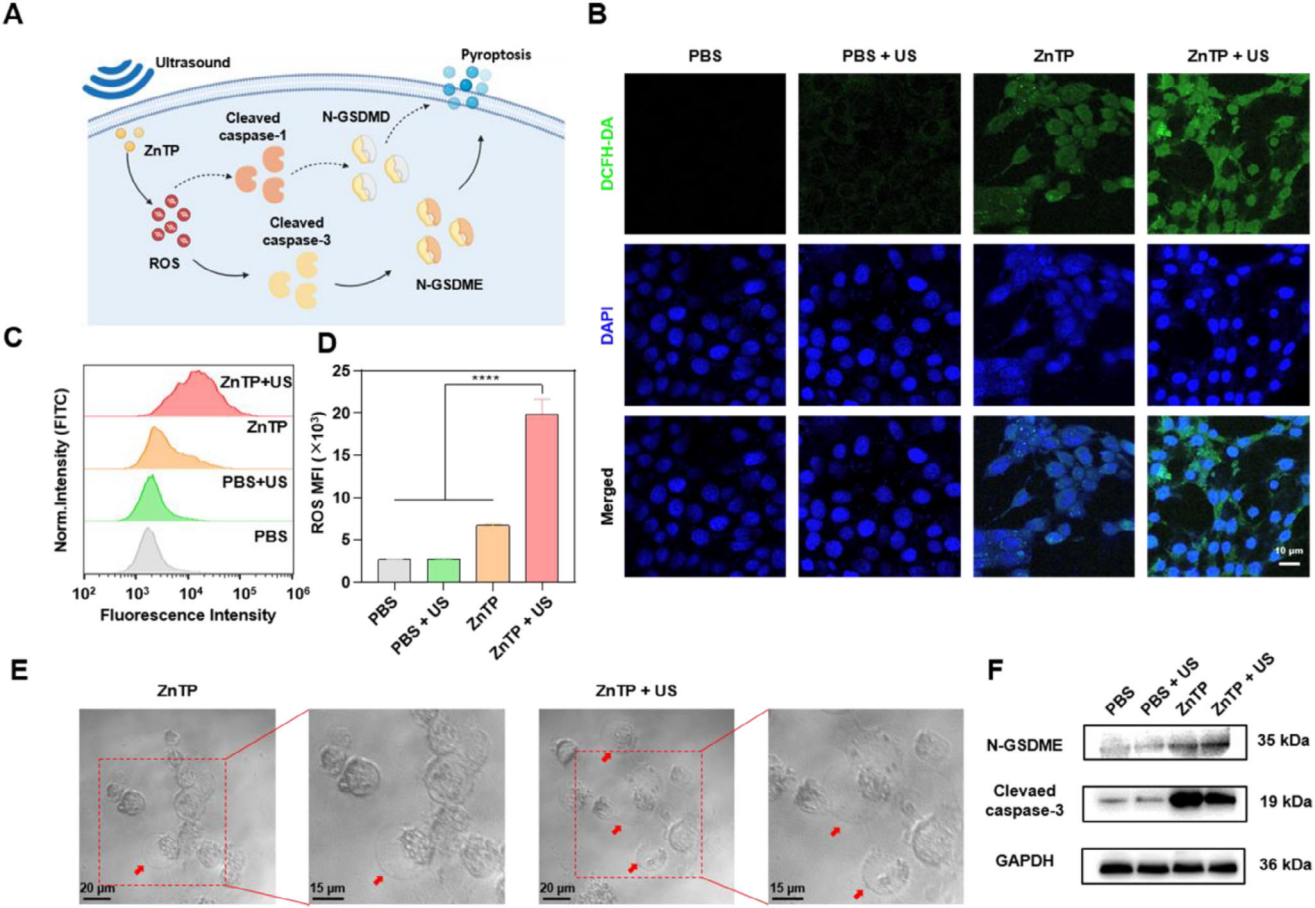
Generation of ROS and induction of pyroptosis by ZnTP upon US irradiation. A) Proposed mechanism of pyroptosis induced by ZnTP upon US irradiation. B) CLSM Images of ROS production in 4T1 cells after treatment with PBS, PBS + US, ZnTP, and ZnTP + US. Green fluorescence indicates the presence of ROS, while blue fluorescence represents DAPI‐stained cell nuclei. C) Quantitative fluorescence analysis of ROS production. D) Levels of ROS production in 4T1 cells after different treatments measured by FCM. Data are presented as mean values ± SD (n = 3). E) Observation of pyroptosis in CAL‐51 cells after various treatments using confocal microscopy. F) Western blot analysis of cleaved caspase‐3 and N‐GSDME protein expression. Statistical significance between each pair of groups was calculated using one‐way ANOVA. ^****^ *p*< 0.0001, indicating a highly significant difference.

The generation of a large amount of ROS and intense inflammatory responses may lead to pyroptosis, prompting our hypothesis that the anticancer mechanism of ZnTP may be associated with pyroptosis (**Figure** **3**A).^[^ ^23^, ^24^, ^25^ ^]^ First, the effect of ZnTP on intracellular ROS production was evaluated using the fluorescent probe 2′,7′‐dichlorodihydrofluorescein diacetate (DCFH‐DA). Observations via CLSM revealed that cells treated with ZnTP + US exhibited significantly higher green fluorescence intensity compared to other groups (Figure 3B). Flow cytometry analysis further confirmed that the fluorescence intensity of cells treated with ZnTP + US was 2.9 times higher than that of cells treated with ZnTP alone (Figure 3C,D). These findings validate that ZnTP + US can effectively generate ROS. Subsequently, the occurrence of pyroptosis was observed through bright‐field microscopy (Figure 3E). Cells treated with ZnTP + US displayed swollen morphologies with membrane blebbing, characteristic of pyroptotic cells.^[^ ^26^, ^27^, ^28^ ^]^

Typically, pyroptosis occurs via two pathways: caspase‐1‐mediated and caspase‐3‐mediated. To investigate the pyroptosis induced by ZnTP + US in detail, Western blot (WB) analysis was conducted (Figure 3F).^[^ ^29^, ^30^ ^]^ The results demonstrated that cells treated with ZnTP + US had an upregulated expression level of cleaved caspase‐3 protein, indicating the activation of caspase‐3. The activated caspase‐3 subsequently cleaved gasdermin E (GSDME), leading to an upregulation of N‐GSDME protein expression. In comparison, cells treated with ZnTP + US did not show activation of cleaved caspase‐1 or gasdermin D (GSDMD) (Figure S10, Support Information). Taken together, these results provide evidence that cells undergo caspase‐3‐mediated pyroptosis following ZnTP + US treatment. In addition, the proportion of lactate dehydrogenase (LDH) release was measured following different treatments. It was found that cells treated with ZnTP + US exhibited the highest LDH release proportion, exceeding 80%. This observation indicates that after ZnTP + US induces pyroptosis in breast cancer cells, membrane rupture is triggered, subsequently leading to the release of cellular contents and consequent elevation in LDH release (Figure S11, Supporting Information).

**Figure 4:**
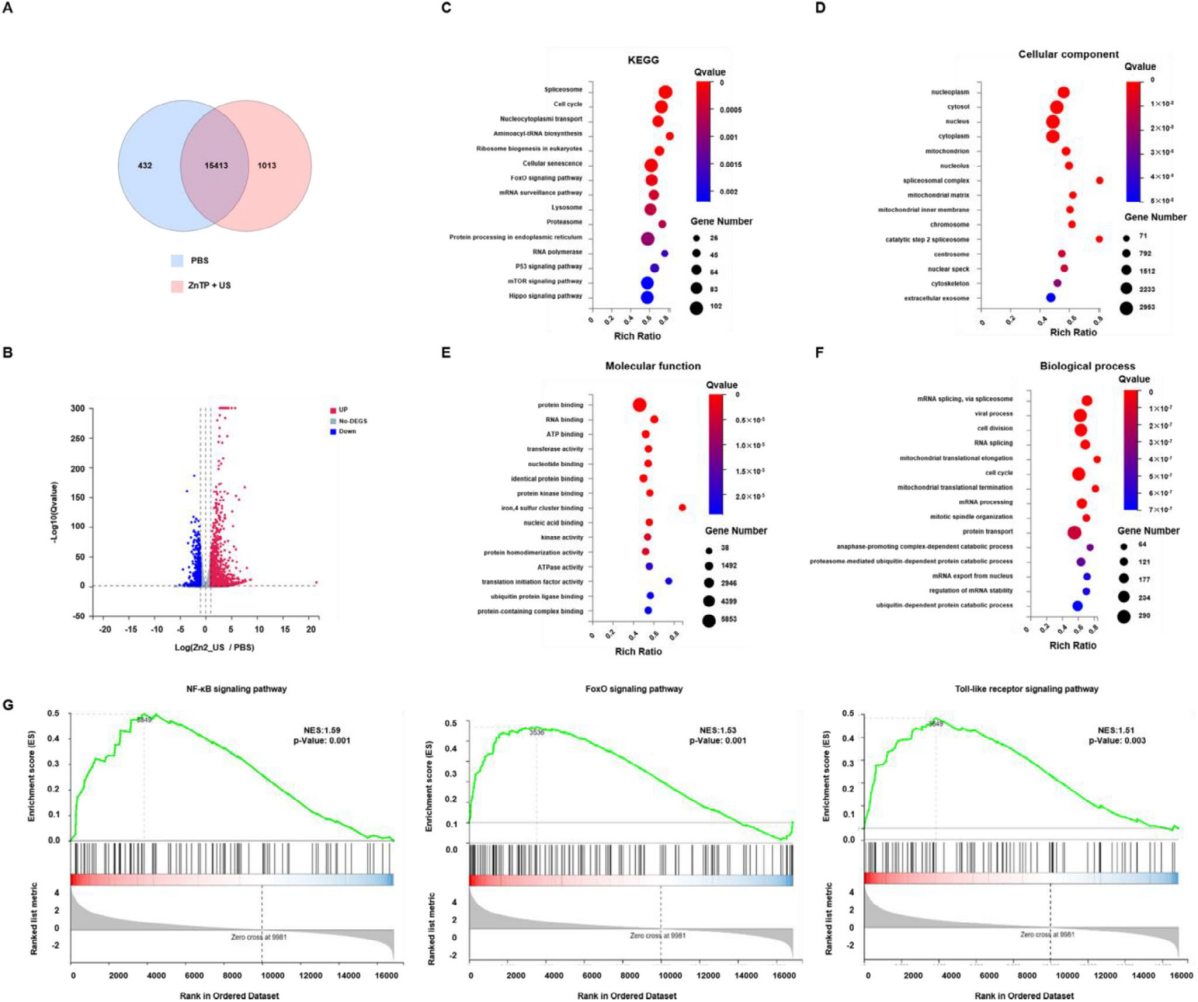
Transcriptome analysis of CAL‐51 cells treated with ultrasound. A) Venn diagrams were constructed to illustrate the identified differentially expressed genes. B) A volcano plot was utilized to display the differential genes that were either upregulated or downregulated in cells treated with ZnTP + US compared cells treated with PBS. C) KEGG analysis was performed on the differentially expressed genes in CAL‐51 cells treated with ZnTP + US. GO categorization of D) cellular components, E) molecular functions, and F) biological processes. G) GSEA analysis revealed gene sets associated with the NF‐κB signaling pathway, FoxO signaling pathway, and Toll‐like receptor signaling pathway. Ultrasonic conditions: 1.0 MHz, 1.0 W cm^−2^, 50% duty cycle, 1 min.

To further elucidate the anticancer mechanisms of ZnTP, RNA sequencing was performed on cells treated with PBS, ZnTP, and ZnTP + US. Venn diagrams revealed significant differences in gene expression levels between cells treated with ZnTP + US and those treated with PBS (**Figure** **4**A). Specifically, among cells treated with ZnTP + US, 1614 genes were upregulated, while 680 genes were downregulated (Figure 4B), compared to cells treated with PBS. Kyoto Encyclopedia of Genes and Genomes (KEGG) analysis indicated that processes such as cytosolic‐nuclear transport and the cell cycle, as well as signaling pathways associated with cytosolic‐nuclear transport, including FoxO signaling, P53 signaling, mTOR signaling, and Hippo signaling, were affected (Figure 4C).^[^ ^31^, ^32^, ^33^, ^34^ ^]^ Gene Ontology (GO) enrichment analysis showed that, in terms of cellular components, gene expression was predominantly affected in components such as the nucleoplasm, cytoplasm, nucleus, cytosol, mitochondria, and nucleolus (Figure 4D).

In terms of molecular functions, binding activities of proteins, ribonucleic acids, adenosine triphosphate, and nucleotides were primarily impacted (Figure 4E). For biological processes, mRNA splicing, cell division, and the cell cycle were predominantly affected (Figure 4F). Gene Set Enrichment Analysis (GSEA) revealed upregulation of a series of inflammation‐related signaling pathway genes, including the NF‐κB signaling pathway, FoxO signaling pathway, and Toll‐like receptor signaling pathway (Figure 4G).^[^ ^35^, ^36^ ^]^ These findings suggest that ZnTP, in combination with ultrasound irradiation, significantly induces inflammatory responses in cancer cells.

**Figure 5:**
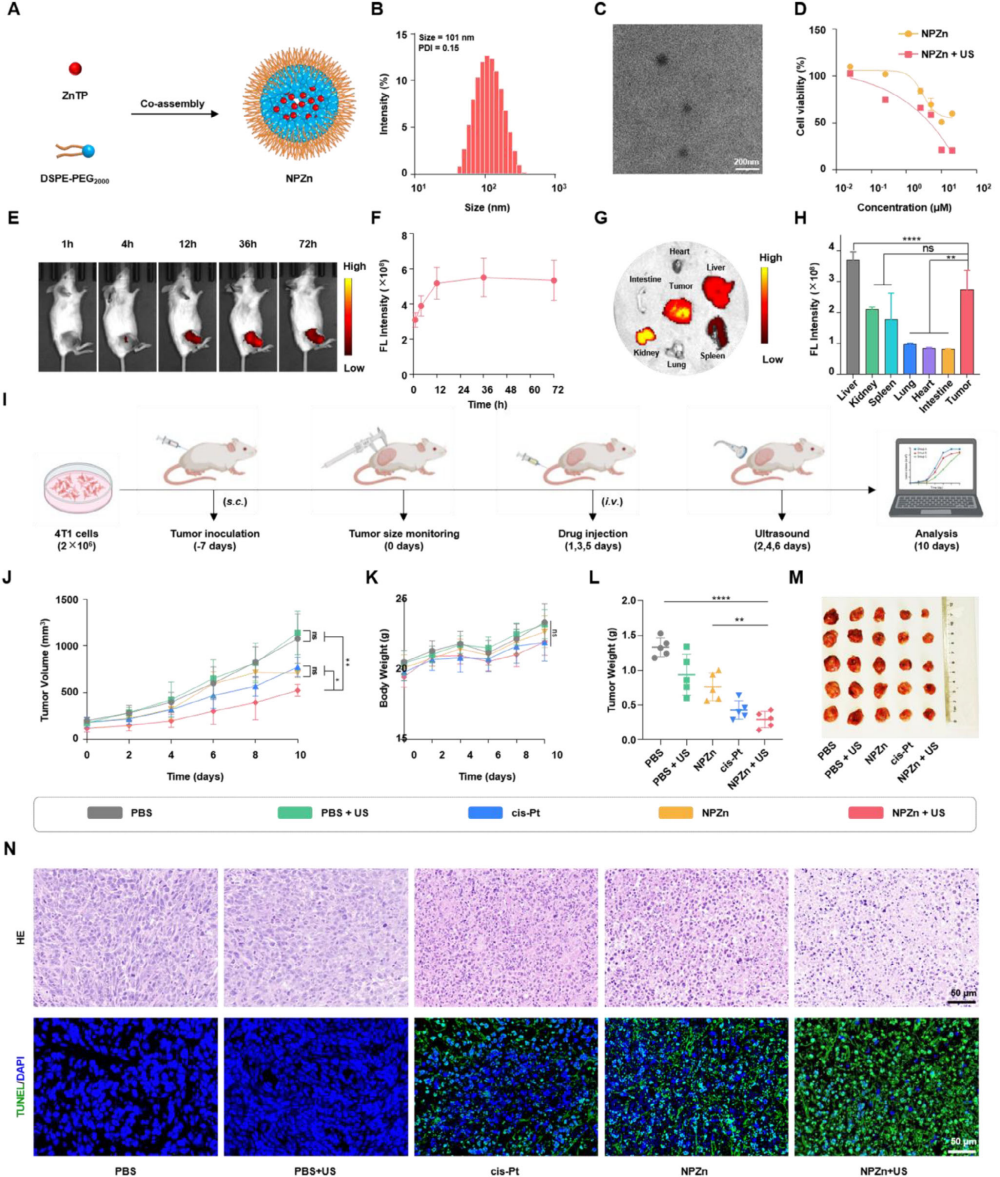
Preparation of NPZn and its in vivo antitumor efficacy under ultrasound irradiation. A) Scheme for the preparation of NPZn. B) Average particle size and polydispersity index (PDI) of NPZn measured by dynamic light scattering. C) Representative TEM image of NPZn. D) Cytotoxicity effect of NPZn. E) Fluorescence imaging of 4T1 tumor‐bearing mice after intravenous injection of NPZn@Cy7.5 (IVIS Spectrum CT, PerkinElmer, E_x_/E_m_ = 745 nm/840 nm). F) Semi‐quantitative analysis of fluorescence intensity at the tumor site at different time points. G) Ex vivo imaging of tumors and major organs (heart, liver, spleen, lungs, kidneys, and intestine). H) Semi‐quantitative analysis of fluorescence intensity in tumors and major organs. I) Therapeutic regimen of NPZn in mice. (Zn: 1 mg kg^−1^, Pt: 1 mg kg^−1^) Ultrasound was applied to the tumor site 24 h after intravenous injection. Ultrasound conditions: 1.0 MHz, 3.0 W cm^−2^, 50% duty cycle, 5 min. J) Comparison of tumor growth inhibition curves. K) Changes in body weight of mice during treatment. L) Tumor mass after different treatments at the end of the study. M) Corresponding tumor images. N) Hematoxylin and eosin staining and TUNEL staining of tumor tissues. Statistical significance between each pair of groups was calculated by two‐way ANOVA followed by Bonferroni's multiple comparison test. ^*^ *p*< 0.05, ^**^ *p*< 0.01, ^***^ *p*< 0.001, ^****^ *p*< 0.0001, ns, not significant. Data are presented as mean values± SD (n = 5).

To improve the solubility of ZnTP, ZnTP was co‐assembled with the commercial amphiphilic polymer DSPE‐PEG_2000_ to form NPZn (**Figure** **5**A). The average diameter of NPZn was measured to be ≈101 nm by dynamic light scattering (DLS), with a polydispersity index (PDI) of 0.15 (Figure 5B). Subsequently, the morphology of NPZn was further observed by transmission electron microscopy (TEM) (Figure 5C). The TEM results indicated an average diameter of 96 nm for NPZn. To evaluate whether NPZn possesses similar anticancer efficacy to ZnTP, the cytotoxicity of NPZn was assessed using the MTT method (Figure 5D). The results showed that the IC_50_ value of NPZn + US was ≈3 µM, demonstrating a cytotoxicity effect comparable to that of the ZnTP + US group, thus justifying the use of NPZn for subsequent animal experiments.

To investigate whether NPZn could accumulate at the tumor site after systemic injection, the in vivo biodistribution of Cy7.5‐labeled NPZn (NPZn@Cy7.5) in 4T1 tumor‐bearing mice was studied using an in vivo imaging system (IVIS Spectrum). Following tail vein injection of NPZn@Cy7.5, the fluorescence signal at the tumor site gradually increased over time and reached a peak at 36 h (Figure 5E,F). After the in vivo imaging, the mice were euthanized, and their major organs and tumor tissues were collected for ex vivo imaging (Figure 5G). The results indicated that the fluorescence intensity in tumor tissue was only lower than that in the liver. This finding suggests that NPZn@Cy7.5 can effectively accumulate at the tumor site.

To evaluate the in vivo antitumor efficacy of NPZn, a subcutaneous 4T1 tumor model was established in BALB/c mice 7 days before the treatment (Figure 5I). On days 1, 3, and 5, mice were injected with PBS, cis‐Pt, and NPZn via the tail vein, and the tumor sites of mice in the PBS + US and NPZn + US groups were subjected to ultrasound treatment (1.0 MHz, 3.0 W cm^−2^, 50% duty cycle, 5 min) 24 h after the treatment. The PBS + US group exhibited similar tumor growth compared to the PBS group, indicating that US treatment alone did not inhibit tumor progression. In contrast, the tumor progression of mice in the NPZn + US group was significantly inhibited, with the highest tumor inhibition rate of 51.3% among all groups. This tumor inhibition rate was 1.8 times that of the cis‐Pt group (28.1%) and 1.5 times that of the ZnTP group(34.1%), demonstrating that NPZn + US exhibits superior antitumor efficacy (Figure 5J). During the treatment period, the body weight of mice in all groups increased steadily, indicating the good biosafety of NPZn (Figure 5K). At the end of the study, the tumor tissues of the mice were collected, and the NPZn + US group showed the smallest tumor mass and size (Figure 5L,M). Additionally, hematoxylin and eosin (H&E) staining revealed more pronounced nuclear fragmentation and nuclear lysis induced by NPZn + US, while terminal deoxynucleotidyl transferase dUTP nick end labeling (TUNEL) staining showed more dead cells in the NPZn + US group (Figure 5N). Therefore, the above results suggest that NPZn + US has higher in vivo antitumor activity than cis‐Pt. Finally, the major organs (heart, liver, spleen, lungs, and kidneys) of mice were subjected to H&E staining to evaluate the biosafety of NPZn. It was observed that all examined organs from NPZn‐treated mice exhibited preserved architectural integrity and normal cellular morphology. Compared with the PBS group, no significant pathological alterations were detected. These collective findings further support the favorable biosafety profile of NPZn at the experimental dose administered (Figure S12, Supporting Information).

**Figure 6:**
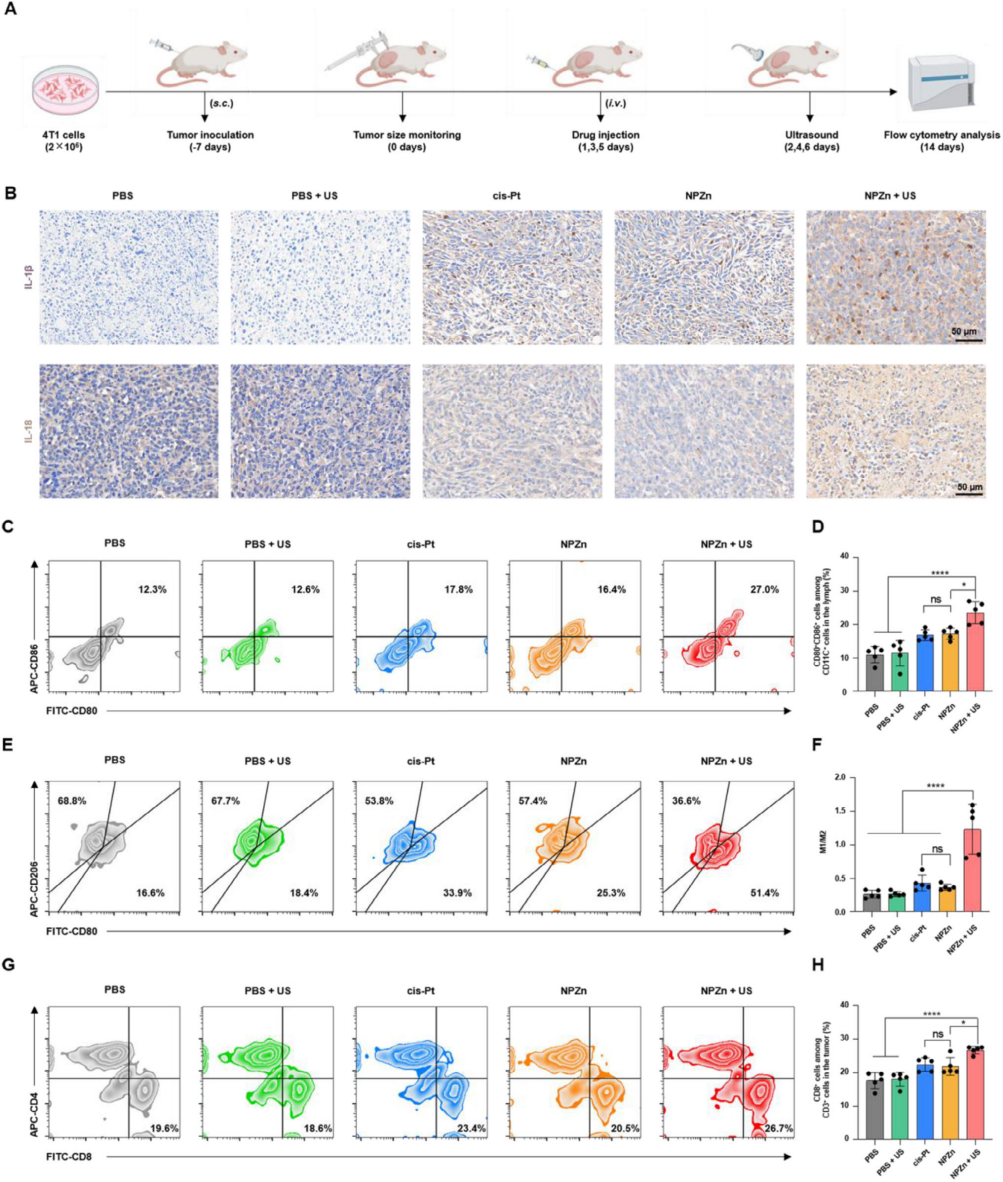
Antitumor immune response induced by NPZn under ultrasound irradiation. A) Schematic treatment schedule of NPZn in 4T1 tumor‐bearing mice. B) Immunohistochemical staining images of IL‐1β and IL‐18 in tumors of mice treated with PBS, PBS + US, cis‐Pt, ZnTP, and NPZn + US, respectively. C) Flow cytometry analysis of CD80^+^CD86^+^ dendritic cells (DCs) within CD11c^+^ cells in the lymph nodes. D) Percentage of CD80^+^CD86^+^ cells among CD11c^+^ cells in the lymph nodes. E) Flow cytometry analysis of CD206^+^ and CD80^+^ cells within tumors. F) Ratio of M1 macrophages to M2 macrophages in tumors. G) Flow cytometry analysis of CD4^+^ and CD8^+^ cells within CD3^+^ cells in tumors. H) Percentage of CD8^+^ cells among CD3^+^ cells in tumors. The data were analyzed by two‐way ANOVA with Bonferroni multiple comparisons post‐test. ^*^ *p*< 0.05, ^****^ *p*< 0.0001, and ns denotes no significant difference. Data are presented as mean values± SD (n = 5).

To investigate the immune activation effect of NPZn in vivo, the lymphoid and tumor tissues of mice from different groups were collected (**Figure** **6**A). The results of immunofluorescence staining have demonstrated that NPZn + US can activate cleaved caspase‐3, inducing pyroptosis (Figure S13, Supporting Information). Pyroptosis is often accompanied by the release of inflammatory cytokines, which subsequently activate the immune response.^[^ ^37^ ^]^ Therefore, the pyroptosis‐associated inflammatory cytokines, IL‐1β and IL‐18, were first investigated through immunohistochemical staining (Figure 6B).^[^ ^38^ ^]^ The results showed that mice treated with NPZn + US showed the highest levels of IL‐1β and IL‐18 in tumor tissues. Combined with the LDH release assay, these experimental outcomes are collectively demonstrated to indicate that inflammatory cell death is induced following NPZn + US treatment. In addition, this finding demonstrated that pyroptosis occurred in tumors of mice treated with NPZn + US, leading to the production of a large number of inflammatory cytokines, which may elicit a strong antitumor immune response.

Furthermore, the immune cell population in lymphoid and tumor tissues was analyzed by FCM. The flow cytometry results revealed the highest proportion of CD80^+^CD86^+^ mature dendritic cell (DCs) (27%) in the lymph nodes of mice treated with NPZn + US, higher than the proportion of mature DCs (20.5%) in mice treated with NPZn alone. This result confirmed that NPZn + US treatment can promote DC maturation (Figure 6C,D). The types of macrophages at the tumor site, including M1 pro‐inflammatory macrophages and M2 anti‐inflammatory macrophages, were further analyzed (Figure 6E). The ratio of M1 to M2 macrophages was the highest (≈1.4) in mice treated with NPZn + US, showing significant differences compared to other groups (Figure 6F). This result indicates that NPZn + US promotes the polarization of macrophages at the tumor site from the immunosuppressive M2 phenotype to the immunostimulatory M1 phenotype. The maturation of DCs and the M1 polarization of macrophages can facilitate T‐cell activation. Therefore, the status of T cells at the tumor site was further investigated (Figure 6G).^[^ ^39^, ^40^ ^]^ In mice treated with NPZn + US, the proportion of CD8^+^ T cells significantly increased to 26.7% (Figure 6H). Notably, mice treated with PBS alone exhibited a low immune response (DC maturation proportion: 12.3%, M1/M2 ratio: 0.24, CD8^+^ T cell proportion: 19.6%), while those treated with PBS + US showed similar effects (DC maturation proportion: 12.6%, M1/M2 ratio: 0.27, CD8^+^ T cell proportion: 18.6%), suggesting that the ultrasound conditions used (1.0 MHz, 3.0 W cm^−2^, 50% duty cycle, 5 min) did not activate immune modulation in vivo.

Additionally, the expression profiles and spatial distributions of CD8 and F4/80 were examined using immunohistochemistry (IHC). Analysis of CD8 IHC revealed that tumor tissues from mice treated with NPZn plus US demonstrated significantly increased infiltration of CD8⁺ T cells, indicating effective T cell activation. Furthermore, evaluation of F4/80 IHC showed that mice treated with NPZn + US exhibited moderately elevated macrophage density relative to mice treated with PBS, with improved infiltration patterns observed within tumor tissues. Collectively, these results provide evidence for the potent anti‐tumor immune response activation mediated by NPZn. (Figure S14, Supporting Information). Overall, the flow cytometry results and IHC results demonstrate that NPZn + US treatment promotes DC maturation and macrophage polarization toward the M1 type, further inducing T‐cell activation and thereby enhancing the antitumor immune response.

ZnTP functions as a highly efficient sonosensitizer, demonstrating superior ROS generation efficiency compared to the clinical small‐molecule sonosensitizer Ce6. Through co‐assembly with DSPE‐PEG_2000_, ZnTP was formulated into NPZn with an average diameter of 101 nm. This nano‐architecture enables enhanced tumor targeting via the EPR effect, resulting in effective tumor accumulation persisting beyond 24 h. Consequently, the therapeutic window for sonodynamic therapy was significantly extended. Mechanistically, NPZn induces tumor cell death through singlet oxygen‐mediated cytotoxicity, activates caspase‐3, and triggers N‐terminal GSDME‐cleavage‐dependent pyroptosis. This inflammatory cell death leads to the release of cellular contents (LDH) and inflammatory cytokines (including IL‐1β and IL‐18), potentiating subsequent anti‐tumor immune responses.^[^ ^41^ ^]^ Critically, whether NPZn establishes long‐term anti‐tumor immunity determines its potential to prevent clinical tumor recurrence. Given NPZn's demonstrated potent anti‐tumor efficacy and capacity to activate anti‐tumor immunity, further investigation is warranted to evaluate its sustained immunotherapeutic effects in future studies.

## 3 Conclusion

In summary, we report the first development of ZnTP, a novel zinc(II) complex exhibiting outstanding efficacy in SDT against breast cancer. ZnTP features a meticulously designed structure that enables efficient intersystem crossing and effective separation of electrons and holes, thereby effectively generating ^1^O_2_ under ultrasound irradiation. The abundant production of ^1^O_2_ significantly activates caspase‐3, inducing the release of N‐GSDME, which subsequently triggers pyroptosis in cancer cells and the release of pro‐inflammatory cytokines IL‐1β and IL‐18. These cytokines further promote DC maturation and M1 polarization of macrophages, effectively activating antitumor immune response and stimulating T‐cell response, synergistically inhibiting the progression of breast cancer. This study not only advanced the molecular design principles for zinc(II)‐based sonosensitizers but also established a solid foundation for their future exploration in SDT.

## 4 Experimental Section

### Reagents and Materials

Unless otherwise noted, all chemicals and reagents were obtained commercially and used without further purification. All reagents were used as received unless otherwise specified.

4′‐Bromo‐2,2′:6′,2′'‐terpyridine,4,4,5,5‐Tetramethyl‐2‐(pyren‐1‐yl)‐1,3,2‐dioxaborolane and DSPE‐PEG_2000_ was purchased from bidepharm, Shanghai, China. MTT and sodium dodecyl sulfate were purchased from Aladdin Co. Ltd. (Shanghai, China). RPMI 1640 medium, DMEM medium, and penicillin/streptomycin (P/S) were purchased from Procell Life Science & Technology Co., Ltd. Propidium iodide (PI), 2‐(4‐amidinophenyl)‐1H‐indole‐6‐carboxamidine (DAPI), and FITC phalloidin were purchased from Solarbio Science & Technology Co., Ltd. (Beijing, China). Annexin V‐FITC/PI cell apoptosis kit, Calcein/PI Live/Dead Viability/Cytotoxicity Assay Kit, bicinchoninic acid (BCA) protein assay kit, and TUNEL apoptosis assay kit were purchased from Beyotime (Shanghai, China). Cleaved Caspase‐3 (Asp175) Antibody (9661) was purchased from Cell Signaling Technology, US. GAPDH Rabbit Monoclonal Antibody (AF1186) was purchased from Beyotime (Shanghai, China). Anti‐cleaved N‐terminal DFNA5/GSDME antibody [EPR20866‐160] (ab222407) was purchased from Abcam. PE anti‐mouse CD11c Antibody, FITC anti‐mouse CD80 Antibody, APC anti‐mouse CD86 Antibody, PE anti‐mouse CD3 Antibody, FITC anti‐mouse CD8 Antibody, APC anti‐mouse CD4 Antibody, APC anti‐mouse CD206 Antibody, and PE anti‐mouse F4/80 were purchased from Biolegend, USA.

### Material Characterization

Nuclear magnetic resonance spectroscopy spectra were obtained by a 400 MHz NMR spectrometer (Bruker) at room temperature. High‐resolution mass spectrometry was recorded on an ESI‐FTICR‐MS. The morphology and size were measured by TEM (Hitachi HT 7700, Japan). The size, Zeta potential, and polydispersity index of the nanoparticles were measured by DLS on an ALV/CGS‐3 goniometry system (ALV, Germany). The UV absorbance spectra were measured using a UV–vis–NIR spectrophotometer (UV‐2600). Quantitative determination of MTT was carried out using a Bio‐Rad microplate reader (SpetraMax M3). Flow cytometric analysis was performed using a FCM (Beckman Coulter, USA). In vivo imaging was conducted by an in vivo imaging System (IVIS, Perkin Elmer, USA). Fluorescence imaging analysis was performed using a CLSM (ZEISS LSM 880, Germany). The samples of the ZnTP, cells, and animals were sonicated by a probe coating with ultrasound (US) gel of portable US apparatus (DJO Chattanooga 2776, USA).

### Synthesis of Compound TP

A mixture of 4′‐Bromo‐2, 2′:6′, 2′'‐terpyridine (300 mg, 0.96 mmol), 4, 4, 5, 5‐Tetramethyl‐2‐(pyren‐1‐yl)‐1, 3, 2‐dioxaborolane (473.2 mg, 1.44 mmol), and Pd(PPh_3_)_4_ (10 mg) in 8 mL toluene, 2 mL ethanol and 8 mL potassium carbonate (2 mol L^−1^) was stirred at 105 °C for 12 h under a nitrogen atmosphere. The reaction mixture was then diluted with dichloromethane (50 mL) and washed with water (20 mL × 3). The organic layer was dried over anhydrous MgSO_4_, filtered, concentrated under vacuum, and purified by silica gel column chromatography (DCM:MeOH = 9:1) to afford compound TP (291.4 mg, yield 70%) as a pale yellow solid. ^1^H‐NMR (400 MHz, CDCl_3_): δ = 8.80–8.74 (m, 4H), 8.70 (d, 2H), 8.25 (m, 3H), 8.19 (d, 1H), 8.13 (t, 3H), 8.09–8.00 (m, 2H), 7.92 (m, 2H), 7.36 (m, 2H).ESI‐FTICR‐MS (m/z):[M + H]^+^ calcd for [434.16572]^1+;^ found[434.16536]^1+^.

### Synthesis of Compound ZnTP

ZnCl_2_ (20.5 mg, 0.15 mmol) dissolved in methanol was added dropwise to a solution of TP(129.6 mg, 0.3 mmol) dissolved in DCM/MeOH(1:1, v/v). The mixture was stirred vigorously overnight at 50 °C under a nitrogen atmosphere protected from light. The precipitate was washed with ether and dichloromethane and subsequently recrystallized by ethanol to give a yellow solid ZnTP (yield 80%).^1^H‐NMR (400 MHz, DMSO‐d_6_): δ = 9.13 (s, 4H), 8.91 (d, 4H), 8.86 (d, 4H), 8.54 (d, 2H), 8.41 (dd, J = 11.3, 4H), 8.36–8.28 (m, 14H), 8.17 (t, 2H), 7.94–7.86 (m, 4H). ESI‐FTICR‐MS (m/z):[M]^2+^ calcd for [465.12192]^2+;^ found[465.12182]^2+^.

### Preparation of NPZn

ZnTP (10 mg) and DSPE‐PEG_2000_ (100 mg) were dissolved in 1 mL of DMSO, which was then added dropwise into 10 mL of water under continuous agitation. Then, the mixture was dialyzed in a dialysis bag (MWCO: 8000–14,000 Da). After 72 h, the mixture was concentrated through ultrafiltration, followed by filtration using a 0.22 µm syringe‐driven filter to give NPZn.

### Preparation of NPZn@Cy7.5

ZnTP (1 mg), Cy5.5 (100 µg), and DSPE‐PEG_2000_ (10 mg) were dissolved in 0.5 mL of DMSO, which was then added dropwise into 10 mL of water under continuous agitation. Then, the mixture was dialyzed in a dialysis bag (MWCO: 8000–14,000 Da). After 72 h, the mixture was concentrated through ultrafiltration, followed by filtration using a 0.22 µm syringe‐driven filter to give NPZn@cy7.5.

### Computational Details

The theoretical calculations were performed via the Gaussian 16 program based on DFT. The structures of the ZnTP were fully optimized using the B3LYP functional and 6–31G(d) basis set. Time‐dependent density functional theory was carried out at the same level. Orbital analysis was carried out by Multiwfn software.

### Reactive Oxygen Species (ROS) Detection Using 1,3‐diphenylisobenzofuran

The ROS generated by ZnTP and Ce6 was measured using DPBF. First, the absorbance of DPBF at 410 nm was adjusted to ≈1.0. Second, the absorbance of ZnTP or Ce6 at 410 nm was adjusted to ≈0.2. Third, the cuvette was exposed to US irradiation(1.0 MHz, 1.0 W cm^−2^, 50% duty cycle) at various times, and absorption spectra were measured immediately.

### Hydroxyl Radical Detection

The hydroxyl radical detection generated by ZnTP was measured using MB. The absorbance of MB at 660 nm was adjusted to ≈1. The concentration of ZnTP was the same as in the previous DPBF test. The absorption spectra of MB were measured immediately every 1 min with a total sonication time of 4 min.

### Electron Spin Resonance Measurements

^1^O_2_ was evaluated by TEMP. 20 µL TEMP (1 M) was mixed with 180 µL ZnTP and irradiated by US (1.0 MHz, 1.0 W cm^−2^, 50% duty cycle, 2 min). The signals of ^1^O_2_ can be shown by the ESR spectrometer. As a comparison, ZnTP without US irradiation was detected too.

### Cell Culture

4T1 cells were cultured in RPMI 1640 medium, and CAL‐51 cells were cultured in DMEM media. Culture mediums were supplemented with 10% (v/v) FBS and 1% (v/v) P/S. All the cell lines were cultured in an incubator at 37 °C containing 5% (v/v) CO_2_.

### Cellular Zinc Uptake

4T1 cells (1 × 10^6^/well) were seeded into 6‐well plates and incubated for 12 h. Then the cells were replaced with fresh medium and added with ZnTP for 1, 4, and 7 h, respectively. The cells were then washed three times with PBS and acidified with nitric acid. Thereafter, Zn content was determined using ICP.

### Cell Viability

Cells were seeded into 96‐well plates (8 × 10^3^ cells/well) and then incubated at 37 °C overnight. Then, the cells were treated with PBS, ZnCl_2_, cis‐Pt, ZnTP with final concentrations of 20, 10, 5, 2.5, 0.25, 0.025 µM for 12 h. Subsequently, the cells were exposed to US irradiation (1.0 MHz, 1.2 W cm^−2^, 50% duty cycle, 2 min). After 24 h, the cell viability was measured by MTT assay. The absorbance of each well was recorded on a microplate reader (Spectra Max) at 570 nm (peak absorbance) and 650 nm (peak background).

### In Vitro Pyroptosis Assays

4T1 cells were seeded into the confocal dishes, incubated overnight, and then treated with PBS, ZnTP (Zn = 10 µM). Subsequently, the cells were exposed to US irradiation (1.0 MHz, 0.5 W cm^−2^, 50% duty cycle, 0.5 min). After being washed with PBS, the morphology of the cells was observed under a confocal microscope. To further verify pyroptosis‐mediated pore formation, 2 × 10^5^ 4T1 cells were seeded into the 24‐well plate, incubated overnight, and then treated with PBS, ZnTP (Zn = 10 µM). Subsequently, the cells were exposed to US irradiation (1.0 MHz, 1.0 W cm^−2^, 50% duty cycle, 1 min). PI staining was performed respectively, and the cell uptake of PI was detected by flow cytometry.

### Colony Formation Assay

4T1 cells were seeded into 6‐well plates (2000 cells/well) and cultured for 12 h. Then, cells were incubated with PBS, ZnCl_2_, cis‐Pt, ZnTP at a concentration of 0.5 µM for 24 h and maintained in RPMI 1640 medium with 10% FBS at 37 °C for 7 days. When the cells grew to visible colonies, they were washed with PBS and fixed with 70% ethyl alcohol for 15 min. Subsequently, the colonized cells were stained with 0.5% crystal violet for another 15 min, and the colonies were photographed with a camera.

### The Live/Dead Cell Staining

For the live/dead state detection, the 4T1 cells were treated with ZnTP (the concentration of Zn was 4 µM) for 12 h. Subsequently, the cells were exposed to US irradiation (1.0 MHz, 1.0 W cm^−2^, 50% duty cycle, 0.5 min). Followed by incubation for 12 h, the 4T1 cells were stained with Calcein AM/PI. Subsequently, images were collected with CLSM.

### Live/Dead Cell Staining of 3D Tumor Spheroids

1% agarose gel solution (50 µL) was added to each 96‐well plate. 2000 4T1 cells (200 µL) were added to each well. On the 7th day, the cell spheres were basically formed. Then the spheroids were treated with PBS, ZnCl_2_, cis‐Pt, ZnTP for 12 h. Subsequently, the cells were exposed to US irradiation (1.0 MHz, 1.0 W cm^−2^, 50% duty cycle, 0.5 min). After being washed with cold PBS, the spheroids were stained with live and dead cell staining assay kits. Subsequently, images were collected with CLSM.

### Intracellular ROS Generation

4T1 cells were incubated with ZnTP (the concentration of Zn was 4 µM) for 24 h, and then mixed withDCFH‐DA before the US irradiation (1.0 MHz, 1.0 W cm^−2^, 50% duty cycle, 1 min). After 30 min, cells were examined by CLSM and FCM to confirm the intracellular ROS.

### Western Blot Analysis

4T1 cells were seeded in 6‐well plates (1 × 10^6^ cells/well) and allowed to adhere overnight. The cells were treated with PBS, ZnTP (Zn = 4 µM) for 24 h. Subsequently, the cells were exposed to US irradiation (1.0 MHz, 1.0 W cm^−2^, 50% duty cycle, 0.5 min). RIPA lysis buffer with protease and phosphatase inhibitors was added to the well. The proteins of cells were extracted through a centrifuge at a speed of 12,000 rpm for 15 min. Protein content quantification was carried out by the BCA protein assay kit. Then, the electrophoreses process was conducted through SDS‐PAGE by a gel‐electrophoretic apparatus (Bio‐Rad mini, USA), and the proteins were transferred to the PVDF films and incubated with the antibodies against various proteins overnight on a shaker at 4 °C. Subsequently, the PVDF films were washed 5 times and incubated with HRP‐conjugated antibodies for 1 h. The Western blot images were obtained using the Amersham Imager 600.

### RNA‐seq Analysis

4T1 cells were treated with PBS, ZnTP (the concentration of Zn was 4 µM) for 12 h. Afterward, the cells were exposed to US irradiation (1.0 MHz, 1.0 W cm^−2^, 50% duty cycle, 1 min), and incubated for another 12 h at 37 °C. Three distinct samples, 2 million cells per sample, from each treatment group, were collected to purify RNA. The RNA quality was confirmed using a NanoDrop 2000/c Spectrophotometer. The sequencing data were submitted to the National Center for Biotechnology Information (NCBI) Sequence Read Archive (SRA) database (Bioproject ID PRJNA1060370), which will be released upon publication. BGISEQ‐500 was employed for sequencing. RSEM was used to quantify the transcription levels of genes.

### Tumor Model and Biodistribution of NPZn@Cy7.5 In Vivo

Female BALB/c mice received a subcutaneous injection of 2 × 10^6^ 4T1 cells at the right flank to build a 4T1 solid tumor‐bearing mouse model. 4T1 tumor‐bearing mice were intravenously injected with NPZn@Cy7.5 followed by imaging with an IVIS Spectrum (PerkinElmer) at 1, 4, 12, 36, and 72 h postinjection, respectively (excitation wavelength: 745 nm, fluorescence emission signal wavelength: 840 nm). 72 h post‐injection, mice were sacrificed to collect tumors and major organs for ex vivo imaging.

### In Vivo Antitumor Efficacy

Tumor‐bearing BALB/c mice were randomly divided into five groups (n = 5): 1) PBS, 2) PBS + US, 3) cis‐Pt, 4) NPZn, 5) NPZn + US (0 days). The mice were injected with various drugs at 1, 3, 5 days. Then at 2, 4, 6 days (24 h after injection), mice in the group PBS + US and NPZn + US were anesthetized and US irradiated (1.0 MHz, 3.0 W cm^−2^, 50% duty cycle, 5 min) at the tumor site. At the designed time (0, 2, 4, 6, 8, and 10 days), the body weight of mice and tumor volume were measured. Ten days later, all mice were sacrificed. The tumors were collected, fixed in 4% paraformaldehyde solution, and then embedded in paraffin for H&E staining and TUNEL staining.

### In Vivo Immune Stimulation

Female BALB/c mice (6 weeks old) were implanted with 4T1 cells (2 × 10^6^) in the right flank. 4T1 tumor‐bearing BALB/c mice were randomly divided into five groups (n = 5): 1) PBS, 2) PBS + US, 3) cis‐Pt, 4) NPZn, 5) NPZn + US (0 days). The mice were injected with various drugs at 1, 3, and 5 days. Then, at 2, 4, 6 days (24 h after injection), mice in groups PBS + US and NPZn + US were anesthetized and US irradiated (1.0 MHz, 3.0 W cm^−2^, 50% duty cycle, 5 min) at the tumor site. Fourteen days later, all mice were sacrificed. The tumors and spleens of mice in each group were harvested, carefully digested, and homogenized into a single‐cell suspension in PBS. The well‐prepared single‐cell suspension was further stained with various fluorescence‐labeled antibodies and analyzed by flow cytometry. For the detection of mature DCs (CD11c^+^CD80^+^CD86^+^), cells collected from lymphocytes were stained with anti‐CD11c‐PE, anti‐CD80‐FITC and anti‐CD86‐APC antibodies. For analysis of M1 and M2 macrophages in the tumor, cells collected from tumors were stained with anti‐F4/80‐PE, anti‐CD80‐FITC, and anti‐CD206‐APC antibodies. For analysis of T cells in tumors, cells collected from tumors were stained with anti‐CD3‐PE, anti‐CD3‐APC and anti‐CD8‐FITC antibodies.

### Statistical Analysis

All obtained data were presented as mean ± standard deviation (SD). Statistical comparisons were made by one‐way or two‐way ANOVA with Bonferroni multiple comparisons post‐test. Statistical significance was defined as ^*^ *p*< 0.05, ^**^ *p*< 0.01, ^***^ *p*< 0.001 and ^****^ *p*< 0.0001. All statistical calculations were carried out with Prism 8 (GraphPad Software).

### Ethical Approval

All animal experiments were conducted in accordance with the guidelines of the Ethics Committee of Peking University (project number: LA2021316).

## Conflict of Interest

The authors declare no conflict of interest.

## Supporting information



Supporting Information

## Data Availability

The data that support the findings of this study are available in the supplementary material of this article.
