# Supplementary material for: A Novel Zinc(II) Complex for Sonodynamic Therapy Induces Pyroptosis of Breast Cancer Cells and Enhances Anti‐Tumor Immune Response
Source: Adv Sci (Weinh). 2025 Oct 21;12(47):e08155. doi: 10.1002/advs.202508155 (PMC12713055; doi:10.1002/advs.202508155)
Supplement: Supplementary file 1 — Supporting Information [file ADVS-12-e08155-s001.docx]

A Novel Zinc(II) Complex for Sonodynamic Therapy Induces Pyroptosis of Breast Cancer Cells and Enhances Anti-tumor Immune Response

Supporting Information

**Figure S1.** Synthetic routes of ZnTP. i) Pd(PPh_3_)_4_, toluene, ethanol, potassium carbonate,105°C, 12 h. ii) DCM, MeOH, 50°C, 12 h.

**Figure S2.** ^1^H NMR spectrum of TP in CDCl_3_.

**Figure S3.** ESI-FTICR-MS of TP.

**Figure S4.** ^1^H NMR spectrum of ZnTP in DMSO-d_6_.

**Figure S5.** ESI-FTICR-MS of ZnTP.

**Figure S6.** UV-Vis absorption (Abs.) spectra of ZnTP.

**Figure S7.** Absorption spectra of MB in the presence of ZnTP under sonication.


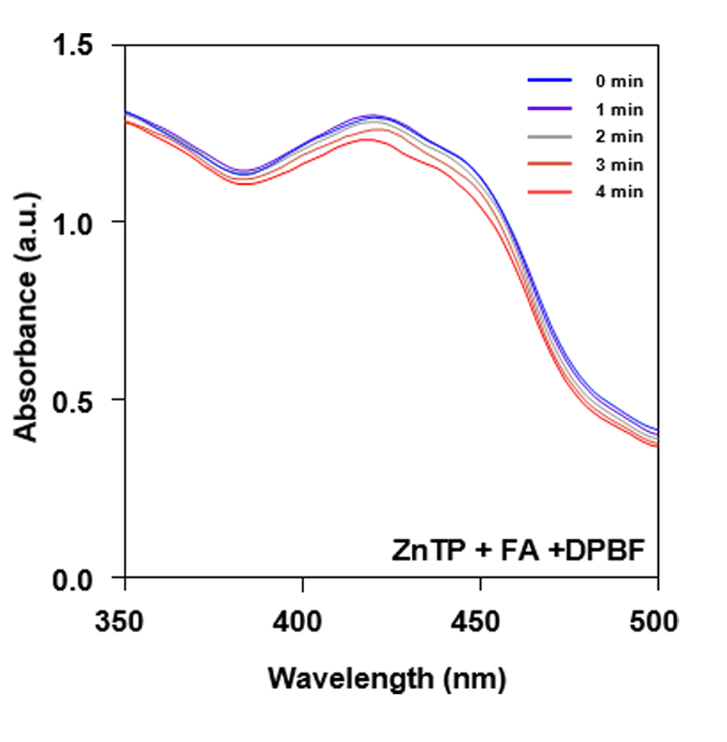


**Figure S8.** Absorption spectra of DPBF in the presence of ZnTP and FA under sonication.


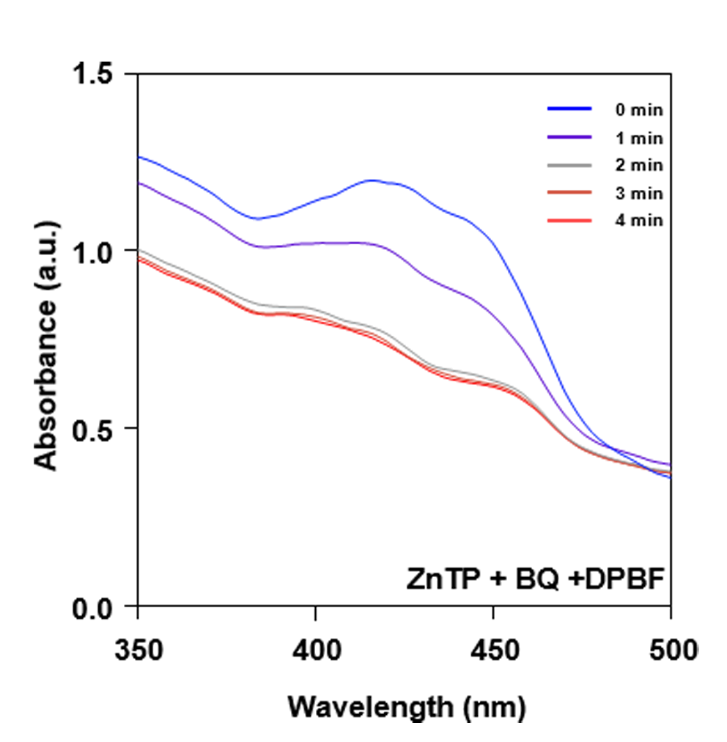


**Figure S9.** Absorption spectra of DPBF in the presence of ZnTP and BQ under sonication.


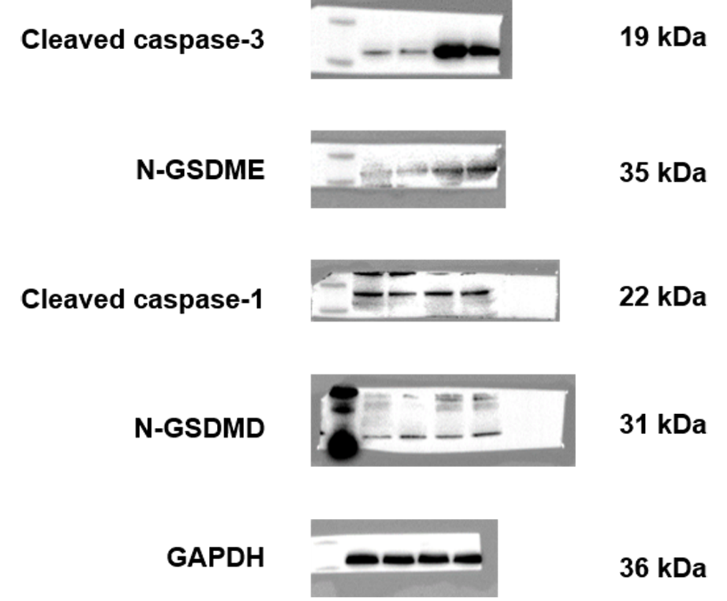


**Figure S10.** Western blot images of cleaved caspas-1，cleaved caspas-3，N-GSDME，N-GSDMD and GAPDH proteins expression after different treatments in 4T1 cells.


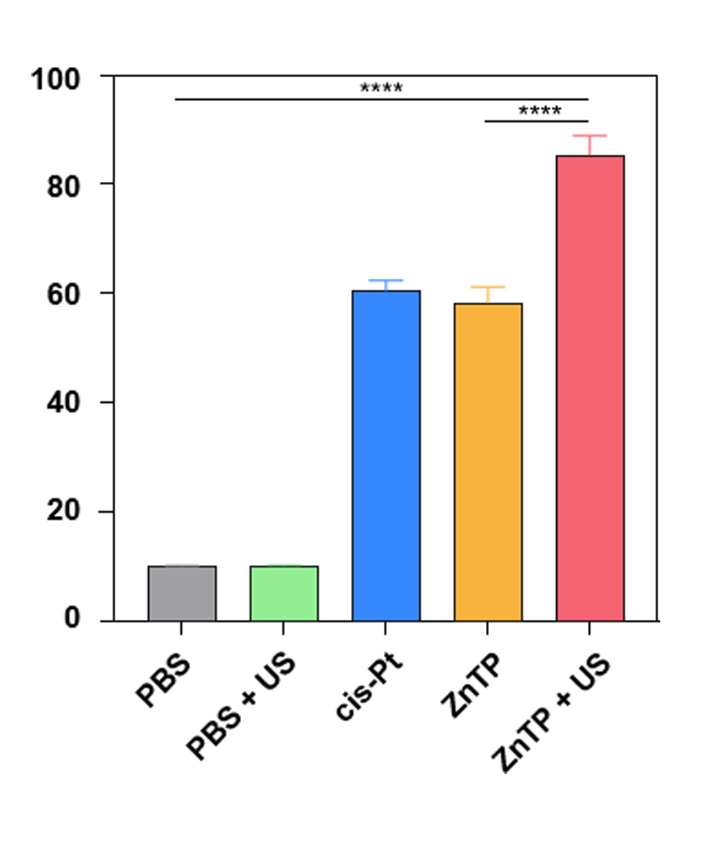


**Figure S11.** Proportion of LDH released in different treatments .


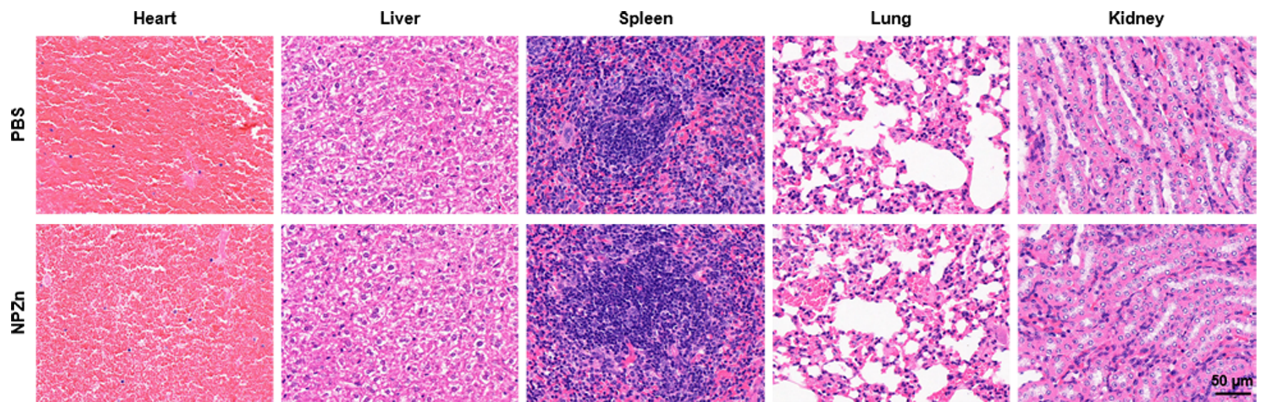
 **Figure S12.** H&E staining images were obtained from mice following administration of PBS or NPZn.

**Figure S13.** A representative immunofluorescent staining images of in a 4T1-tumor-bearing mouse model upon exposure to ultrasonic irradiation. DAPI (blue) and cleaved caspase-3 (red).


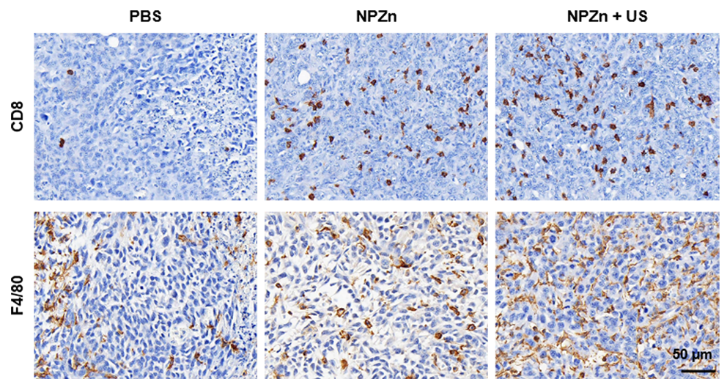


**Figure S14.** Immunohistochemical (IHC) analysis was performed to detect CD8 and F4/80 expression in tumor tissues obtained from mice treated with PBS, NPZn, or NPZn + US.
